# Human monkeypox virus: An updated review

**DOI:** 10.1097/MD.0000000000030406

**Published:** 2022-09-02

**Authors:** Nawal Adnan, Zargham ul Haq, Asmara Malik, Asim Mehmood, Uzma Ishaq, Maria Faraz, Jahanzeb Malik, Amin Mehmoodi

**Affiliations:** a Department of Medicine, Jinnah Sindh Medical University, Karachi, Pakistan; b Department of Medicine, Lahore Medical and Dental College, Lahore, Pakistan; c Department of Community Medicine, National University of Medical Sciences, Rawalpindi, Pakistan; d Department of Medicine, Shifa Tameer e Millat University, Islamabad, Pakistan; e Department of Hematology, Healthways Laboratories, Rawalpindi, Pakistan; f Department of Project Management, Bahria University, Islamabad, Pakistan; g Department of Electrophysiology, Armed Forces Institute of Cardiology, Rawalpindi, Pakistan; h Department of Medicine, Ibn e Seena Hospital, Kabul, Afghanistan.

**Keywords:** orthopoxvirus, outbreak, pandemic, public health, smallpox

## Abstract

The human monkeypox is an emerging zoonotic orthopoxvirus with a clinical presentation similar to that of smallpox. It is difficult to differentiate monkeypox from other orthopedic infections, and laboratory diagnosis is the primary component of disease identification and monitoring. However, current diagnostics are time-consuming, and new tests are needed for rapid and precise diagnosis. Most cases have been reported in Central Africa; however, an increasing number of cases have been reported in Europe, the United States of America (USA), Australia, and the United Arab Emirates. Although investigation of the current global outbreak is still ongoing, viral transmission seems to have occurred during crowded events in Spain and Belgium. New therapeutics and vaccines are being deployed for the treatment and prevention of monkeypox, and more research on the epidemiology, biology, and ecology of the virus in endemic areas is required to understand and prevent further global outbreaks.

## 1. Introduction

Monkeypox is an emerging zoonotic disease in humans that arises from an orthopoxvirus belonging to the Poxviradea family, which is known to have a complex double-stranded DNA.^[1,2]^ Human monkeypox infection is observed in smallpox posteradication areas. Monkeypox virus has a propensity to spread among mammals, including humans. The natural host of the monkeypox virus remains largely unknown, but it has been isolated from a wild animal, once from a ropy squirrel in the Democratic Republic of Congo and once from a sooty mangabey in Côte d’Ivoire.^[3]^ The incubation period of the monkeypox virus, as seen in human-to-human transmission, is 12 days.^[4]^

It is believed that the virus is transmitted through respiratory secretions and saliva, or through direct contact with the exudate or crust material of the lesion. Viral shedding through feces is another potential source for the transmission of the virus.^[1]^

Monkeypox virus has morphologic features similar to other orthopoxviruses, with a size of 200–250 nm, a brick-shaped virus that is enveloped and contains surface tubules along with a dumbell-shaped core component.^[5]^ The central region of the genome of the monkeypox virus is 96.3% similar to that of the variola virus, which codes for structural proteins and essential enzymes, and differs substantially from the region of the genome that codes for virulence factors and host range factors.^[6]^ The 3.4 to 10% case fatality rate of monkeypox lies between the case fatality rates of variola minor and variola major, which have case fatality rates of 1% and 30%, respectively.^[1]^

The disease is indigenous to the Democratic Republic of Congo, where the first case was reported in 1970.^[3]^ However, numerous cases of monkeypox have been reported in humans and wildlife in Central and West Africa.^[1]^ The number of cases of human monkeypox virus has surged in recent years along with an increase in the geographic spread of the disease, as immunity to smallpox vaccination is waning.^[7,8]^ In 2017, Nigeria experienced the largest outbreak of monkeypox virus in the West African clade with a 6% fatality rate.^[3,9,10]^ Two cases of monkeypox were imported to the United Kingdom (UK) through Nigerian individuals in September 2018, and 1 became the source of nosocomial infections affecting healthcare workers.^[11]^ In addition, the monkeypox virus was imported to the United States of America (USA) in 2003 through rodents that were shipped from Ghana and housed with prairie dogs, which became the source of infection in humans.^[5,10,12,13]^

**Figure 1.**
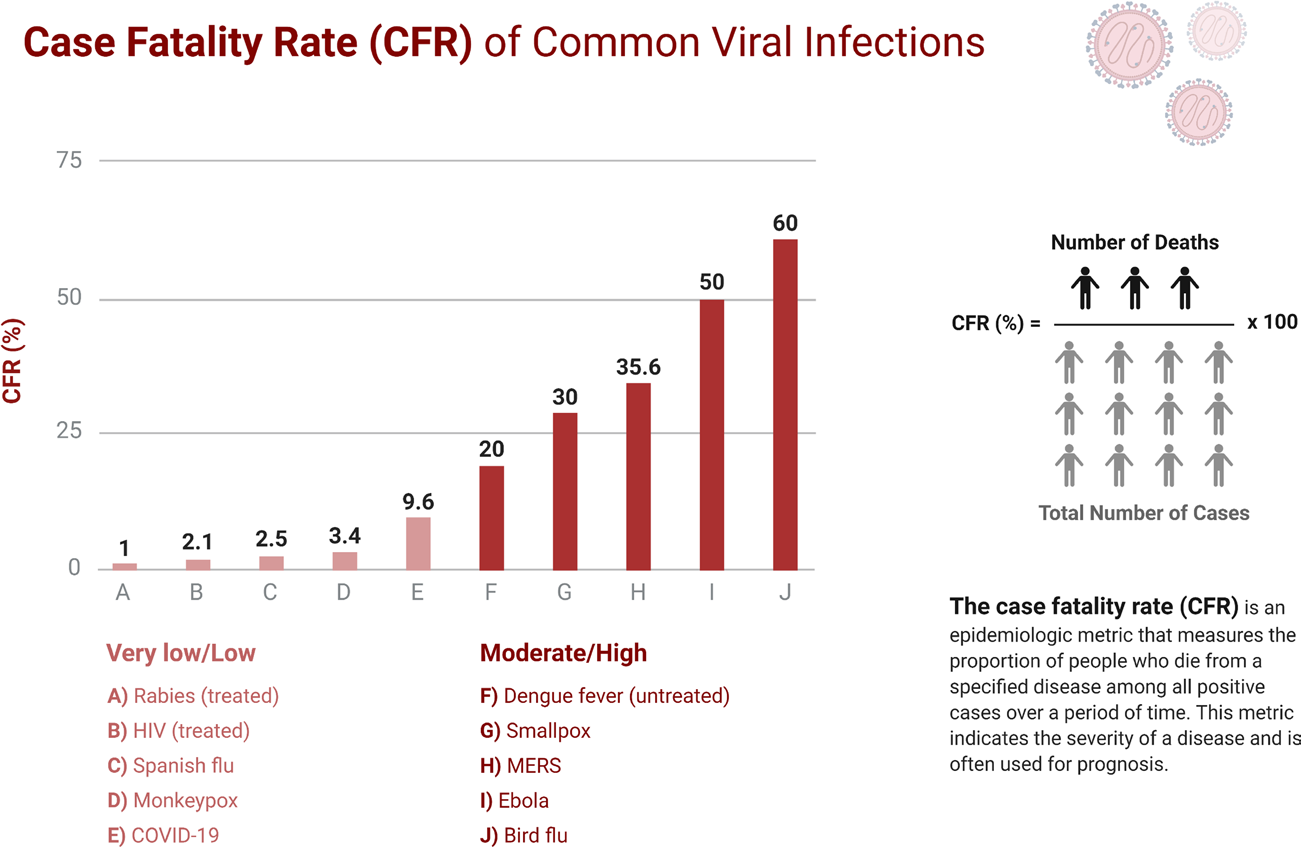
Case-fatality rates of common viral infections. (Adapted from “Case fatality rates of common viral infections”, by biorender.com).

Two clades of monkeypox viruses have been identified: the West African clade and the Central African clade. The former has a case fatality rate of <1 percent with no report of human-to-human transmission, whereas the latter has a case fatality rate of 11%, and human-to-human transmission has been documented in the Central African clade. The virus that was involved in the US outbreak was considered the West African variant, and the one that involved the Democratic Republic of Congo was the Central African variant.^[12,13]^ It has been observed that the Central African clade, also known as the Congo basin clade, is associated with increased morbidity and mortality, human-to-human transmission, and viremia as compared to the West African clade.^[5,10,13]^ Moreover, in the US, adults were disproportionately affected, contrary to the norm, and no fatalities were reported.^[12]^ In Africa, 1.5 to 17% of mortality is recorded, predominantly in children, due to the unavailability of medical care.^[12]^ Figure 1 shows the fatality rates of common viral infections. Although human-to-human transmission of the Western clade was not observed in the USA, in Nigeria, the infection was transmitted through human-to-human interaction, despite being a West African clade.^[10]^

It has been demonstrated that the majority of the cases of monkeypox occurred in children <10 years of age. The attack rate was noted to be significantly higher in individuals who had no previous vaccination mark (7.2%) as compared to the attack rate for individuals who had a vaccination mark. (0.9%).^[14]^ The case fatality rate is 11% in individuals not vaccinated against smallpox because the vaccine offers cross-protection against the monkeypox virus.^[3]^

Despite having little propensity to spread among humans, the monkeypox virus poses a serious threat to life in the region of the Democratic Republic of Congo, West and Central Africa, and possibly globally.^[1]^ High risk is associated with the monkeypox virus, as it can cause a disease of public health significance.^[1]^ In 1980, the World Health Organization (WHO) identified the monkeypox virus as the most important orthopoxvirus infection in humans after the eradication of smallpox; hence, surveillance is warranted.^[14]^ Although there is no evidence currently indicating that human-to-human transmission can sustain monkeypox virus in local communities, a study suggests that repeated exposure to animals infected with monkeypox in a population with low herd immunity can result in large clusters of individuals infected with monkeypox virus in the African rainforest.^[4]^ Similar to smallpox, the monkeypox virus poses a potential threat to biological warfare; hence, various antivirals and therapeutic medications are being developed.^[15]^

## 2. Methods

Scopus, MEDLINE, and Web of Science databases were searched using various medical subject heading (MeSH) combinations to identify the studies of interest. Following MeSH headings were used: “Monkeypox” OR “Human monkeypox” OR “Monkeypox virus” AND “Public health” OR “Outbreak” OR “Clinical features” OR “Epidemiology”. The information gathered from the included studies was incorporated into a narrative style for an evidence-based and updated report on the human monkeypox virus.

### 2.1. Main text

#### 2.1.1. Epidemiology.

The monkeypox virus has 2 clades, the Congo Basin and West Africa, with each causing disease; however, the West African clade is considered less virulent.^[16,17]^ The monkeypox virus has demonstrated infectivity among a wide variety of multiple mammalian species, and contrary to its name, it has never been isolated from any species of monkey, having only been isolated once from a wild animal, a Funisciurus squirrel in the Democratic Republic of Congo. In light of this, the WHO is considering measures to rename the disease to avoid stigma, similar to the renaming of COVID-19.^[18]^ Further research is needed to identify the exact reservoir of the virus and its circulation in the wild.^[19]^

Human infections have been linked to contact with wild animals, but the precise exposure mechanism has been difficult to triangulate, particularly in areas where contact with animals may have occurred through household rodent infestations or hunting or preparation of bushmeat for domestic consumption. Transmission has been shown to occur via saliva and respiratory excretions, or contact with lesion exudate, crust material, or feces of infected animals. Previous evidence indicates that household members or those who care for a monkeypox patient are at an increased risk of acquiring an infection; however, it is less efficient than that observed in smallpox. Household members who were not vaccinated for smallpox showed a greater risk of acquiring the infection than those who were not vaccinated.^[20]^

The first case of human monkeypox was reported in 1970 in West Africa. Subsequently, most reported infections have occurred in the Congo Basin of Central Africa. Mortality varies between 3–6% and 17%, depending on the clade, and children, pregnant women, and immunocompromised individuals are at a high risk for adverse outcomes.^[16]^

**Figure 2.**
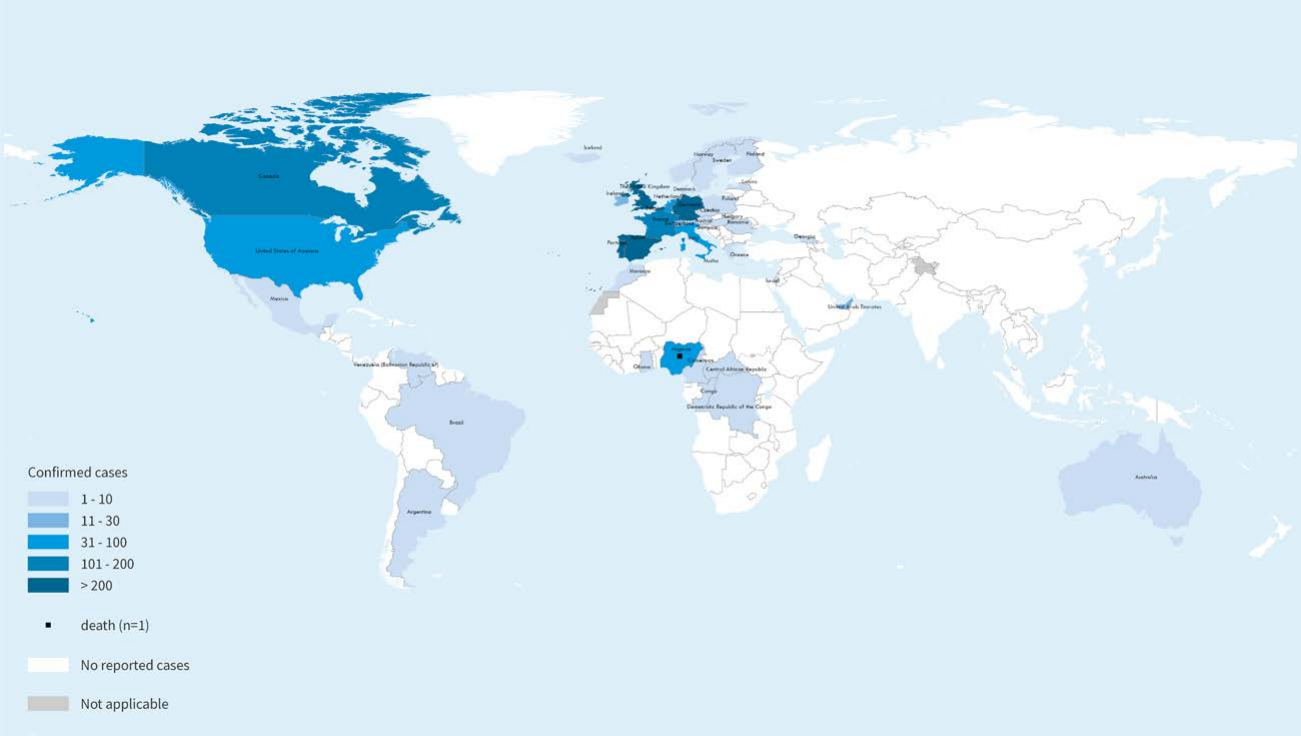
Geographic distribution of monkeypox. Reference: World Health Organization (June 17,2022). Disease Outbreak News; Multi-country monkeypox outbreak in nonendemic countries: Update. Available at: https://www.who.int/emergencies/disease-outbreak-news/item/2022-DON393 (By permission of WHO).^[21]^

As of June 17, a total of 2103 laboratory-confirmed cases and 1 probable case, including 1 death, had been reported to the WHO (Fig. 2). Most reported cases in the recent outbreak have presented through sexual health or other health services in primary or secondary healthcare facilities, with a history of travel primarily to countries in Europe, the USA, or other countries rather than to countries where the virus was not historically known to be present, and increasingly recent travel locally or no travel at all. The outbreak of monkeypox continues to primarily affect men who have sex with men who have recently reported sex with new or multiple partners. At present, the WHO has assessed the risk at the global level as moderate considering that this is the first time that many monkeypox cases and clusters have been reported concurrently in many countries in widely disparate WHO geographical areas; however, mortality has remained very low in the current outbreak.^[22]^

#### 2.1.2. Pathophysiology.

**Figure 3.**
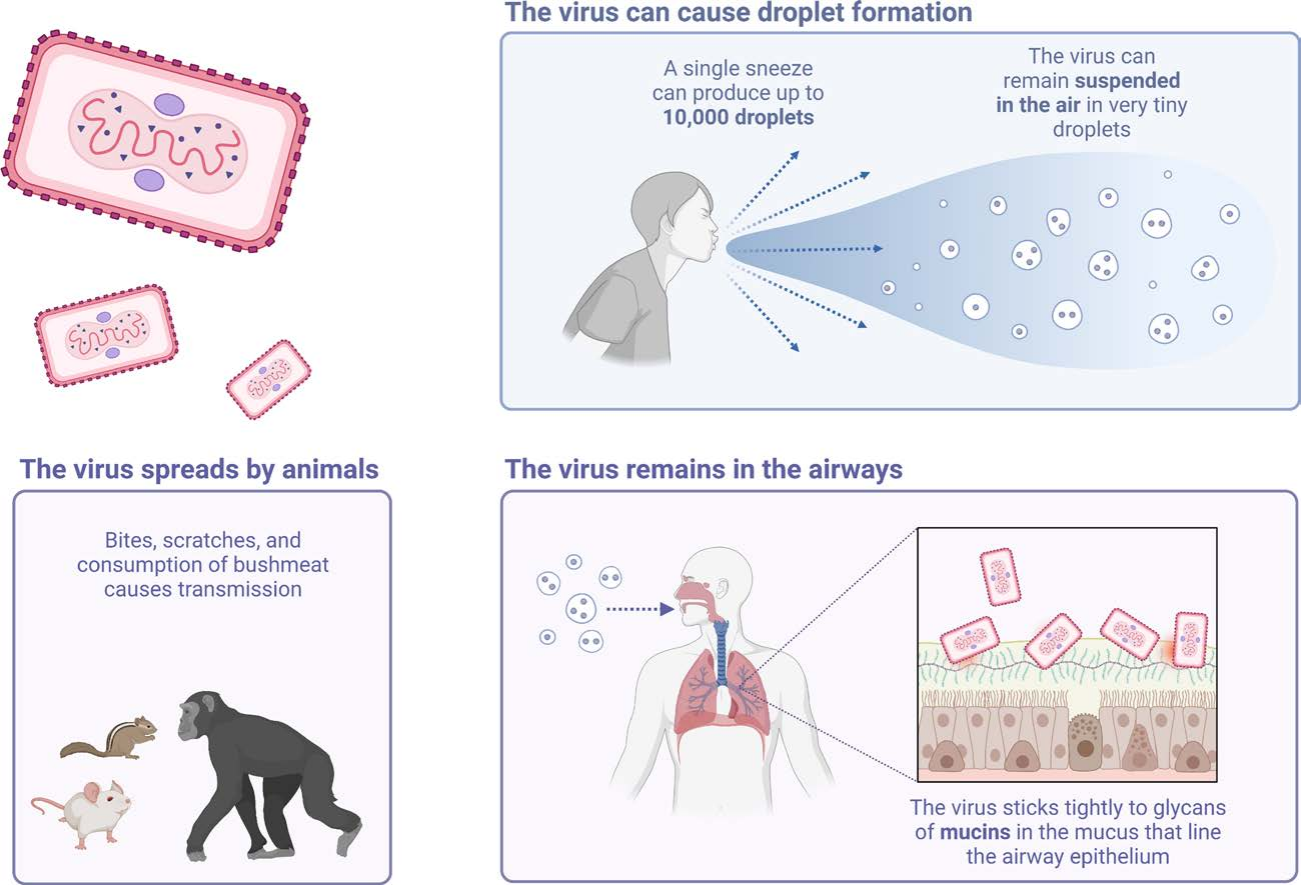
How is the virus spread? (Created with biorender.com).

The monkeypox virus transmission cycle commences with the virus infecting the respiratory epithelium, after which it spreads through the lymphomatous route to infect the major systemic organs and replicate there, indicating primary viremia (Fig. 3). During this stage, little to no virus was detectable in the blood, because the virus was efficiently removed by the reticuloendothelial system of the body. Primary viremia is followed by secondary viremia, which results when the virus is released from the infected organs and lymphoid tissues in the blood and reaches the cornified layer of the skin and mucosal epithelium to give rise to rash and mucosal lesions, respectively. It is to be further noted that the severity of the exanthem and enanthem is largely dependent on a load of virion in the bloodstream during secondary viremia.^[5]^

Patients with smallpox infections have lesions that are confluent and contain an abundant amount of fluid in the vesicular and pustular phases, which are collected in the hypodermic region and then ooze out in the crusting phase. It is worth mentioning that during the transition of these phases, shock may occur owing to the depletion of massive intravascular volume.^[12]^ Similarly, in patients with monkeypox infection in the US, those who reported symptoms of mucosal and gastrointestinal issues needed volume replacement secondary to gastrointestinal fluid loss.^[12]^ The underlying mechanism for volume repletion is the movement of fluid from the intravascular compartment to the extravascular compartment due to hypoalbuminemia and fluid loss in the gastrointestinal tract, as observed in systemic infections. This is evidence that monkeypox infection results in systemic compromise, and the complications are not limited to mucosal and integumentary surfaces, as is apparent by the clinical presentation of the disease.^[12]^

In an experimental model, monkeypox virus was aerosolized into monkeys and viremia was observed, after which the virus spread to disseminated lymph nodes, spleen, thymus, skin, oral mucosa, gastrointestinal tract, and the reproductive system through a lymphogenous route.^[12]^

While studying the pathophysiology of smallpox, it was revealed that individuals with the most dangerous form of smallpox-hemorrhagic smallpox were likely to have disseminated intravascular coagulation. However, 2 US patients from the monkeypox outbreak, who were reported to have hemorrhagic pustules, had no evidence of disseminated intravascular coagulation, but mild thrombocytopenia was noted.^[12]^

#### 2.1.3. Clinical picture.

The clinical features of monkeypox infection are quite similar to those of smallpox, but there are some distinguishing features attributed to monkeypox, such as the enlargement of lymph nodes occurring with the onset of fever early in the course of the disease.^[1]^ Pronounced lymphadenopathy is a hallmark of monkeypox.^[12]^ Almost 90% of individuals affected with monkeypox virus show lymphadenopathy, which can occur unilaterally or bilaterally, affecting the submandibular, cervical, postauricular, axillary, or inguinal lymph nodes, either singly or in combination.^[6]^ Lymph nodes that were enlarged were firm, tender, and occasionally painful.^[23]^ The size of the enlarged lymph nodes was approximately 1 to 4 cm, similar to the size of a pigeon’s egg.^[7]^

However, the course of this disease is milder than that of smallpox.^[12]^ The dominant clinical features attributed to monkeypox infection, as seen in patients after the monkeypox outbreak in the US, included rash, fever, chills, adenopathy, headache, and myalgia.^[7,12]^ These symptoms were observed during the initial presentation of illness. Some individuals reported symptoms of tonsillitis with or without pharyngitis, and coughing was also common.^[7]^ The average duration of fever was reported to be 8 days, while that of the rash was 12 days, and the average period from the onset of fever until the onset of rash was noted to be 2 days; however, this period was as long as 12 days in some patients.^[12]^ Nausea and vomiting are possible findings in the second week of illness, resulting in severe dehydration.^[23]^ Table 1 shows the differences between the clinical pictures of smallpox and monkeypox.

**Table 1:** Clinical features of monkeypox and smallpox.

Features	Monkeypox	Smallpox
Time		
Incubation	7–14 days	7–14 days
Prodrome	Upto 4 days	Upto 4 days
Rash period	14–30 days	14–30 days
Symptoms		
Fever	Yes	Yes
Headache	Yes	Yes
Tiredness	Yes	Yes
Lymphadenopathy	Yes	No
Rash distribution	Centrifugal	Centrifugal
Rash characteristics	Hard, first maculopapular, then vesicular and pustular, well-circumscribed	Hard, first maculopapular, then vesicular and pustular, well-circumscribed
Rash progression	Slow progression from 1 stage to the other; every stage lasting 2–3 days	Slow progression from 1 stage to the other; every stage lasting 2–3 days

**Figure 4.**
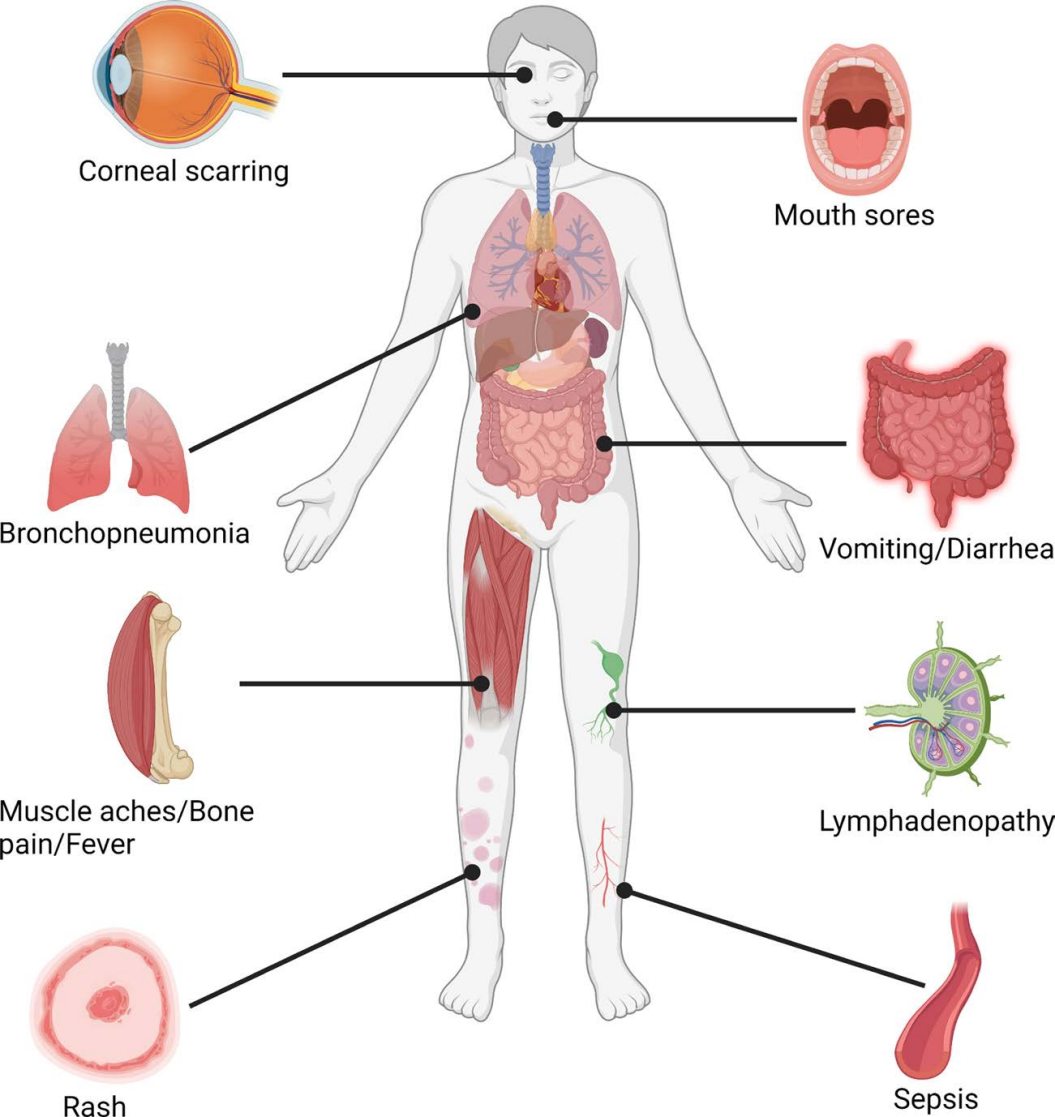
Clinical features of human monkeypox infection. (Created with biorender.com).

One to 3 weeks after the onset of fever and lymphadenopathy, a rash appears, with lesions that erupt simultaneously and evolve at a similar rate. The rash has a peripheral distribution, but can envelop the entire body in the setting of severe disease.^[1]^ The rash was monomorphic with a centrifugal distribution.^[12]^ The rash progresses from the macular stage to the papular stage and then to the vesicular and pustular stage; eventually, the pustules would take an umbilicated form, would scab, and desquamate, indicating termination of the rash.^[5,7,16]^ Individuals infected with human monkeypox may report a few to thousands of lesions during the rash phase. It was also observed that some lesions were present in the oral cavity, making it difficult for patients to eat and drink.^[23]^ The size of the lesions is described as being 0.5 to 1 cm in diameter.^[5,7]^ In addition, few lesions were reported to be ulcerated or necrotic, and even fewer lesions (2 US patients) were noted to have hemorrhagic pustules.^[12]^ Individuals with a previous vaccination history of smallpox reported mild rash that was pleomorphic, lymphadenopathy was present in roughly half of these individuals, and no mortality was reported.^[23]^ In individuals who survive the illness, postinfection scarring is one of the most common long-term sequelae.^[23]^ Figure 4 highlights the major complications associated with monkeypox virus infections in humans.

The duration of the infection was approximately 4 weeks, and desquamation of the lesion suggested that the infection was in the ending phase. Patients can suffer from a host of complications during this viral illness such as respiratory distress, secondary bacterial infection, gastrointestinal involvement, bronchopneumonia, sepsis, dehydration, corneal infection resulting in loss of vision, and encephalitis.^[1]^ Complications were more prevalent in individuals who were not previously vaccinated (74%) than in those who were vaccinated (39.5%).^[23]^ Abnormal laboratory findings were noted in the setting of this viral illness, including elevated transaminase levels, low blood urea nitrogen levels, hypoalbuminemia, leukocytosis, and thrombocytopenia. It took a period of 3 to 12 days from the onset of illness to examine abnormal laboratory findings.^[12]^

Pediatric patients (individuals under 18 years old) are prone to be admitted to an intensive care unit; however, they are not at an increased risk of developing severe illness.^[12]^ One pediatric patient in the US outbreak underwent mechanical ventilation due to encephalitis, while the other suffered from a massive retropharyngeal abscess and cervical lymphadenopathy, which compromised the tracheal airway, both of which were treated in the intensive care unit.^[12]^

Approximately one-third of adults in the US infected with monkeypox were vaccinated against smallpox; however, no significant difference was observed in the clinical presentation of vaccinated and unvaccinated individuals.^[12]^ In the US, patients who had dysphagia and hypoxemia and presented with mouth sores were more likely to have severe illness.^[12]^

Often, a misdiagnosis of varicella is made in monkeypox infection because it is also a febrile rash. However, some distinguishing clinical features can distinguish these 2 diseases. Varicella rash is centripetal, unlike that of monkeypox, which has a centrifugal pattern of distribution. The lesions of varicella appear superficially with irregular borders similar to a “dew drop on a rose petal”; however, the lesions of monkeypox infection are deep and hard, with well-circumscribed margins and umbilication. The rash has a rapid progression in varicella with lesions in multiple stages of development on the body, whereas the monkeypox rash progresses slowly, and the lesions are in a similar stage of development throughout the body. Moreover, lymphadenopathy is not observed in varicella and is a telltale sign of monkeypox infection.^[23]^

The prognosis of human monkeypox infection can be estimated by considering whether the individual has presented with complications and analyzing a few factors, including the health and previous vaccination status of the individual and the presence of any illness such as malaria, measles, or diarrheal disease occurring concomitantly with monkeypox infection.^[7]^

### 2.2. Diagnosis and treatment

Diagnosis of monkeypox infection is made by subjecting the material collected from the lesion to various techniques, such as electron microscopy and immunohistochemistry, to observe the presence of the monkeypox virus. Techniques such as real-time PCR can also be used to trace the virus in a sample, as they are highly effective and sensitive for the detection of viral DNA. Serological markers such as antiorthopoxvirus IgM and IgG can also be used to assess previous exposure to an orthopoxvirus, either through infection or vaccination.^[23]^ Since most cases of monkeypox virus are endemic in rural areas where electricity is not available, advances should be made to develop assays that can be tested in a very basic setting with limited training of personnel.^[23]^ Table 2 lists the various techniques used to diagnose monkeypox infection.

**Table 2:** Diagnostic tests for *Orthopoxvirus.*

Test	Characteristics
Viral cultures	A live virus is grown and it can be helpful in the classification of the species but needs a specimen from lesions
	The assay takes several days to obtain results and can be disrupted by bacterial infiltration
	Needs highly skilled technical staff and major laboratory setting
Immunohistochemistry	This tests for specific *Orthopoxvirus*-specific antigens Any biopsy specimen can be used for detection
	Classification is not possible as *Orthopoxviruses* are indistinguishable from each other
	Needs highly skilled technical staff and major laboratory setting
Real-time polymerase chain reaction	This tests for monkeypox-specific DNA signatures and it can determine previous exposure and the specific monkeypox virus
	Needs highly skilled technical staff and major laboratory setting
Anti- *Orthopoxvirus*immunoglobulins (IgM/IgG)	This tests for*Orthopoxvirus*antibodies to assess a recent or previous exposure
	This test can be used even with smallpox vaccination
	Requires a cold chain and collection of blood and the essay is not specific for monkeypox
Immunohistochemistry	This tests for *Orthopoxvirus*-specific antigens
	Needs highly skilled technical staff and major laboratory setting

At present, no evidence-based treatment for monkeypox infection exists; symptomatic treatment and supportive care are the only ways to manage the patient.^[1]^ Several compounds show therapeutic potential against orthopoxvirus species, including cidofovir, which is used against a range of viruses as it exerts its effect through inhibition of DNA polymerase. Since cidofovir causes nephrotoxicity, a modified form of this drug-CMX-001, an oral antiviral medication, without the adverse effect of cidofovir, is in the stages of development and acts by inhibiting DNA polymerase.^[23]^ Another orally administered drug, ST-246, inhibits the release of intracellular viruses and has shown encouraging results against different species of orthopoxviruses, including variola virus. It has also successfully treated the monkeypox virus in nonhuman primates and small mammals.^[5,23]^

Since the smallpox vaccine confers cross-protection against monkeypox, a single dose of a third-generation smallpox vaccine called Imvanex was offered as postexposure prophylaxis as an off-label indication to individuals at intermediate and high risk of becoming infected with monkeypox virus in the UK in 2018.^[17]^ Recent developments in nanotechnology have been observed in medical research, where metal-based nanoparticles are used in biological systems. Many questions regarding this sophisticated technique remain unanswered. Nevertheless, silver-containing nanoparticles with a 10 nm diameter (Ag-PS-10) proved to be the most effective at inhibiting monkeypox virus infectivity, as observed by the dramatic reduction in monkeypox plaque formation at all concentrations tested.^[15]^

## 3. Public health implications

The reemergence of monkeypox infection in the wake of healthcare system disruptions caused by the COVID-19 pandemic points to many areas of public health, particularly in countries with limited resources. Although smallpox vaccination provides partial immunity against monkeypox infection, its global cessation after the eradication of smallpox disease puts many younger people at risk of infection. Indeed, most of the present cases have been reported in individuals under the age of 50 years. However, long-lasting immunity (>25 years) against monkeypox infection has been demonstrated in communities vaccinated against smallpox in the DRC.^[20]^ This also indicates a glaring double-standard against the global concern for this disease. While monkeypox has remained an endemic problem in Central and West African countries, having limited healthcare resources for monitoring and surveillance of this disease, as well as facing disruption in the form of war and climate change, it has only been given global attention once it infected countries in the Global North. The emergence of COVID-19 has led to large-scale deforestation, migration, conflict, climate change, and the increasing encroachment of the human population into previously untouched habitats may create grounds for the reemergence of more diseases and even push their mutation into more virulent strains of previously benign infections, which may render our current therapeutic interventions and vaccinations redundant. Countries in the Global North must share better diagnostic tools and research with monkeypox-endemic countries to create better engagement with local communities. Once a more comprehensive One Health approach is implemented, research on vaccines and therapeutic agents for these reemerging zoonotic diseases will be more productive and allow for the scaling up of prevention strategies.^[22]^

## 4. Conclusion

The human monkeypox is an infection with the potential to spread via zoonotic reservoirs. The most important reason for the displacement of the virus in nonendemic areas seems to be civil conflict and the relaxation of international travel. Other causes include the movement of people in the natural habitats of animal reservoirs for this virus, which makes humans prone to interaction with wildlife and zoonoses. The latest outbreak in the incidence of human diseases requires further investigation to better understand the range of factors associated with disease transmission and spread. Epidemiological risk factors, animal reservoirs, and COVID-19 coinfection should be the focus of current investigations in endemic areas. Control and preventive measures, including education and personal cleanliness, should be implemented at all incident locations worldwide. Monkeypox should be included in standard disease monitoring protocols, with periodic genomic studies and serological surveys, and health professionals and high-risk populations should be evaluated for vaccination. Because monkeypox is no longer a rare disease, it is necessary to identify animals in Africa that harbor orthopoxviruses, develop a better algorithm for diagnosing the monkeypox clinical spectrum and disease severity, and assess the risk of transmission concerning various types of contact with clinical cases.

## References

[1] Sklenovská N Van Ranst M. Emergence of monkeypox as the most important orthopoxvirus infection in humans. Front Public Health. 2018;6:241. 30234087 10.3389/fpubh.2018.00241 PMC6131633

[2] Jezek Z Marennikova SS Mutumbo M. Human monkeypox: a study of 2,510 contacts of 214 patients. J Infect Dis. 1986;154:551–5. 3018091 10.1093/infdis/154.4.551

[3] Huhn GD Bauer AM Yorita K. Clinical characteristics of human monkeypox, and risk factors for severe disease. Clin Infect Dis. 2005;41:1742–51. 16288398 10.1086/498115

[4] Durski KN McCollum AM Nakazawa Y. Emergence of Monkeypox − West and Central Africa, 1970-2017. MMWR Morb Mortal Wkly Rep. 2018;67:306–10. 29543790 10.15585/mmwr.mm6710a5 PMC5857192

[5] Di Giulio DB Eckburg PB. Human monkeypox: an emerging zoonosis. Lancet Infect Dis. 2004;4:15–25. 14720564 10.1016/S1473-3099(03)00856-9 PMC9628772

[6] McCollum AM Damon IK. Human monkeypox. Clin Infect Dis. 2014;58:260–7. 24158414 10.1093/cid/cit703 PMC5895105

[7] Hutin YJ Williams RJ Malfait P. Outbreak of human monkeypox, Democratic Republic of Congo, 1996 to 1997. Emerg Infect Dis. 2001;7:434–8. 11384521 10.3201/eid0703.010311 PMC2631782

[8] Likos AM Sammons SA Olson VA. A tale of two clades: monkeypox viruses. J Gen Virol. 2005;86:2661–72. 16186219 10.1099/vir.0.81215-0

[9] Reynolds MG Damon IK. Outbreaks of human monkeypox after cessation of smallpox vaccination. Trends Microbiol. 2012;20:80–7. 22239910 10.1016/j.tim.2011.12.001

[10] Yinka-Ogunleye A Aruna O Ogoina D. Reemergence of Human Monkeypox in Nigeria, 2017. Emerg Infect Dis. 2018;24:1149–51. 29619921 10.3201/eid2406.180017 PMC6004876

[11] Jezek Z Szczeniowski M Paluku KM. Human monkeypox: clinical features of 282 patients. J Infect Dis. 1987;156:293–8. 3036967 10.1093/infdis/156.2.293

[12] Vaughan A Aarons E Astbury J. Human-to-human transmission of monkeypox virus, United Kingdom, October 2018. Emerg Infect Dis. 2020;26:782–5. 32023204 10.3201/eid2604.191164 PMC7101111

[13] Beer EM Rao VB. A systematic review of the epidemiology of human monkeypox outbreaks and implications for outbreak strategy. PLoS Negl Trop Dis. 2019;13:e0007791. 31618206 10.1371/journal.pntd.0007791 PMC6816577

[14] Shchelkunov SN Totmenin AV Safronov PF. Analysis of the monkeypox virus genome. Virology. 2002;297:172–94. 12083817 10.1006/viro.2002.1446 PMC9534300

[15] Yinka-Ogunleye A Aruna O Dalhat M. CDC Monkeypox Outbreak Team. Outbreak of human monkeypox in Nigeria in 2017–18: a clinical and epidemiological report. Lancet Infect Dis. 2019;19:872–9. 31285143 10.1016/S1473-3099(19)30294-4 PMC9628943

[16] The Lancet Infectious Diseases. Monkeypox: a neglected old foe. Lancet Infect Dis. 2022;22:S1473–3099. 10.1016/S1473-3099(22)00377-2 PMC9628760 35691304

[17] Erez N Achdout H Milrot E. Diagnosis of Imported Monkeypox, Israel, 2018. Emerg Infect Dis. 2019;25:980–3. 30848724 10.3201/eid2505.190076 PMC6478227

[18] Taylor L. Monkeypox: WHO to rename disease to prevent stigma. BMJ[Internet]. 2022;377:o1489. Available at: https://www.bmj.com/content/377/bmj.o1489 [access date June 18, 2022]. 10.1136/bmj.o1489 35710105

[19] WHO. Monkeypox [Internet]. World Health Organisation. Available at: https://www.who.int/news-room/fact-sheets/detail/monkeypox [access date June 18, 2022].

[20] Moore M Zahra F. Monkeypox. In: StatPearls [Internet]. Treasure Island, FL: StatPearls Publishing, 2022. Available at: https://www.ncbi.nlm.nih.gov/books/NBK574519/ [access date May 18, 2022].

[21] World Health Organization. Disease Outbreak News; Multi-country monkeypox outbreak in non-endemic countries: Update. Jun 2022. Available at: https://www.who.int/emergencies/disease-outbreak-news/item/2022-DON393

[22] Multi-country monkeypox outbreak: situation update [Internet]. Available at: https://www.who.int/emergencies/disease-outbreak-news/item/2022-DON393 [access date June 18, 2022].

[23] Heymann DL Szczeniowski M Esteves K. Re-emergence of monkeypox in Africa: a review of the past six years. Br Med Bull. 1998;54:693–702. 10326294 10.1093/oxfordjournals.bmb.a011720

